# A redeemed strategy for molecular autopsy in unexplained infant deaths

**DOI:** 10.1186/s12967-024-05130-w

**Published:** 2024-04-02

**Authors:** Yuqi Yang, Haixin Li, Jingyu Zhao, Hui Huang, Bin Yu

**Affiliations:** 1 Department of Medical Genetics, Changzhou Maternal and Child Health Care Hospital, Changzhou Medical Center of Nanjing Medical University, No. 16 Ding Xiang Road, Changzhou, Jiangsu Province China; 2 Clinical Lab, BGI Genomics, Nanjing, 210019 China; 3 BGI Genomics, Shenzhen, 518083 China

## Dear Editor:

The causes of child death are very complex and the diagnosis remains a significant challenge. Even through extensive investigation, about 10.5% of deaths cannot be clearly explained [1]. Unexplained deaths have become a leading cause, greatly blocking the scientific guidance of re-birth. Autopsy is considered an accurate method for investigating unexplained deaths. However, it is not acceptable for most parents, with less than 50% of cases being carried out due to complex reasons, such as religious and cultural beliefs [1]. As a result, a considerable number of cases could not get a definite etiological diagnosis of child death even after investigation. Recently, molecular autopsy have been reported to have great value in revealing the cause of unexpected death, including autopsy-negative cases. It could explain approximately 12.6%∼44.0% of previously unexplained deaths with DNA sequencing [2, 3]. However, opportunities for molecular autopsy are often lost due to the lack of timely sample collection.

Newborn screening (NBS), an important public health program, is widely used throughout the world and covers almost all newborns. The stored samples of neonatal dried blood spots (DBS) from NBS are considered valuable resources for medical research. DNA extracted from DBS can be used to diagnose and study genetic diseases [4]. Here, we attempt to remedial molecular autopsy for child death using clinical exome sequencing (CES) based on the stored DBS.

From August 2019 to August 2023, a total of 444 child deaths were recorded in the surveillance system, which collected the occurrence and distribution of under-5 child deaths. There are 35 categories of causes of death in the system. 36 cases of unexplained deaths were included as the subjects, including 8 cases with an unknown diagnosis and 28 cases with listed causes that remained doubtful during the basic death survey. The time of their deaths ranged from 3 days to 48 months. Within this cohort, 6 (16.67%) were newborns (0–28 days), 14 (38.89%) were infants (1–11 months) and 16 (44.44%) were children (12–59 months). We collected the stored DBS from their previous NBS.

3 ∼ 8 dried blood spots with a diameter of 3 mm were taken, and DNA extraction was performed using the MagPure DNA KF Kit B and KingFisher Flex. When the DNA concentration exceeded 1ng/µL, follow-up experiments were conducted. The library was constructed by capturing the target area independently developed by BGI Genomics Co., Ltd, with a quality control standard set at > 20 ng/µL. DNA nanoball libraries were generated by rolling circle amplification of circularized DNA libraries and sequenced on the MGISEQ-2000 sequencer platform (MGI) using a paired-end 100 bp plus 10 bp (index) strategy with an average depth of ≥ 180-fold. The bioinformatics analysis process, including data filtering, alignment, mutation detection, and result annotation, was performed as previously described [5].

32 cases successfully received effective results based on the stored DBS, yielding a CES success rate of 88.9%. However, four cases failed due to DNA quality not meeting the standard after library construction. CES identified disease-related genetic variation in 6 out of 32 child, including 4 cases of SNVs and 2 of CNVs. The abnormal detection rate was 18.8% (95% CI, 7.2-36.4%) (Table 1). In detail, one child (case 1) was diagnosed as LIG4 syndrome with a compound heterozygous for pathogenic (P) and like pathogenic (LP) variants in the *LIG4* gene (c.833G > T and c.2141T > A). He has sought medical attention multiple times due to immune deficiency and recurrent respiratory infections. The boy was suspected of LIG4 syndrome when he was two years old, but there was no definitive diagnosis. After one year, he underwent allogeneic hematopoietic stem cell transplantation but died postoperatively due to ineffective treatment for bacteremia and graft-versus-host reaction. Case 2, despite long-term treatment and rehabilitation, has been experiencing persistent epileptic seizures since birth, and the cause was unclear. CES detected an LP heterozygous variant (c.107G > A) in the *CPA6* gene associated with familial temporal lobe epilepsy 5, which follows an autosomal dominant inheritance pattern. Case 3 showed global developmental delay during growth and development with cranial MRI revealing slightly larger bilateral ventricles, less white matter, and a thinner epiphyseal body. CES analysis identified compound pathogenic heterozygous variants in the *GLB1* gene (c.1343 A > T and c.1063 C > T), linked to GM1 gangliosidosis/β-galactosidase deficiency 1. The boy (case 4) was diagnosed with malignant tumors in both eyes shortly after birth, undergoing consecutive chemotherapy sessions but experiencing a worsening condition. He died at the age of 2 years and 10 months. The pathogenic variant c.607 + 1G > T was detected in the *RB1* gene. Case 5 and case 6 were reported pathogenic CNVs, involving a microdeletion (0.49 Mb) in the 11q23.3 region for case 5 and a microduplication (3.59 Mb) in 13q33.3-q34 for case 6. These segments include a certain number of coding genes. Further data analysis identified pathogenic genes related to the death phenotype. For example, case 5 died due to congenital acute lymphoblastic leukemia, while the *KMT2A* gene (11q23.3 region) is closely associated with mixed type leukemia.

**Table 1:** Positive results found by ES based on DBS

Single Nucleotide Variation (SNV)												
Case	Gender	Death time	Gene	Chr	cHGVS	pHGVS	Exon	Zygosity	Mode	Type	Phenotype	Disease
Case 1	Male	3 years	*LIG4*	chr13	c.2141T > A	p.Leu714*	EX2E	Het	AR	LP	Congenital immunodeficiency	LIG4 syndrome (OMIM:606,593)
					c.833G > T	p.Arg278Leu	EX2E	Het		P		
Case 2	Male	2 years	*CPA6*	chr8	c.107G > A	p.Arg36His	EX1	Het	AD/AR	LP	Status epilepticus	Familial temporal lobe epilepsy 5 (OMIM:614,417)
Case 3	Female	2 years	*GLB1*	chr3	c.1343 A > T	p.Asp448Val	EX13	Het	AR	P	Congenital brain injury	GM1 gangliosidosis/β-galactosidase deficiency 1 (OMIM:230,500)
					c.1063 C > T	p.Gln355*	EX10	Het		P		
Case 4	Male	2 years	*RB1*	chr13	c.607 + 1G > T		IVS6	Het	AD	P	Malignant tumors in both eyes	Retinoblastoma (OMIM:180,200)
**Copy Number Variation (CNV)**												
**Case**	**Gender**	**Death time**	**Result**				**Include coding genes**				**Phenotype**	**Disease**
Case 5	Male	28 days	46,XN, del(11q23.3).seq[GRCh37/hg19](118,359,328–118,851,946)*1				*ARCN1 DDX6 KMT2A(intron10-3’UTR) TREH, etc.*				Leukemia	*KMT2A* gene is associated with mixed type leukemia.
Case 6	Male	3 years	46,XN, dup(13q33.3-q34).seq[GRCh37/hg19](107,220,767–110,807,744)*3				*LIG4 COL4A1 IRS2, etc.*				Brain tumor	*COL4A1* gene is associated with autosomal dominant pons microvascular disease.

Moreover, some minor findings are also worth paying attention to. Although currently interpreted as variants of uncertain significance (VUS), a total of nine cases (28.1%; 95% CI, 13.7-46.7%) were detected with disease-related genetic variations (Table 2).

**Table 2:** Minor findings of this study

Single Nucleotide Variation (SNV)												
Case	Gender	Death time	Gene	Chr	cHGVS	pHGVS	Exon	Zygosity	Mode	Type	Phenotype	Disease
201,818,593	Male	3 years	*GLDC*	chr9	c.3049 A > G	p.Arg1017Gly	EX25E	Het	AR	VUS	Brainstem encephalitis	Nonketotic hyperglycinemia (OMIM:605,899)
					c.2136 C > G	p.Asp712Glu	EX18	Het		VUS		
201,835,756	Female	4 years	*ACTN1*	chr14	c.143 C > T	p.Ala48Val	EX2	Het	AD	VUS	Disseminated Intravascular Coagulation, DIC (Unknown causes)	Bleeding disorder, platelet-type, 15 (OMIM:615,193)
201,935,603	Female	1 years	*POT1*	chr7	c.1802 C > T	p.Pro601Leu	EX19E	Het	AD	VUS	Pineal region tumor	Tumor predisposition syndrome 3 (OMIM:616,568)
202,011,946	Female	1 years	*SHOC2*	chr10	c.365T > C	p.Leu122Ser	EX2	Het	AD	VUS	Atrial septal defect	Noonan syndrome-like with loose anagen hair 1 (OMIM:607,721)/AD
202,023,508	Male	2 years	*MYLK*	chr3	c.5141T > G	p.Leu1714Arg	EX31	Het	AD	VUS	Acatalepsy	Aortic aneurysm, familial thoracic 7 (OMIM:613,780)
202,101,256	Female	8 days	*PRNP*	chr20	c.233G > C	p.Gly78Ala	EX2E	Het	AD	VUS	Acatalepsy	Creutzfeldt-Jakob disease (OMIM:123,400) Insomnia, fatal familial (OMIM:600,072)
202,106,290	Male	1 months	*RNF213*	chr17	c.6802 C > T	p.His2268Tyr	EX29	Het	AD/AR	VUS	Vascular malformations of the brain? (Uncertain causes)	{Moyamoya disease 2, susceptibility to} (OMIM:607,151)
202,116,626	Female	2 years	*ABCC9*	chr12	c.285-7T > C	-	IVS2	Het	AD	VUS	Cardiopulmonary arrest (Unknown causes)	Cardiomyopathy, dilated, 1O (OMIM:608,569)
202,205,085	Female	24 days	*KLHL40*	chr3	c.554delG	p.Gly185Alafs*14	EX1	Het	AR	LP	Cardiopulmonary arrest (Unknown causes)	Nemaline myopathy 8, autosomal recessive (OMIM:615,348)
					c.523G > A	p.Asp175Asn		Het	AR	VUS		

Undoubtedly, identifying the cause of child death holds great significance for reproduction and reducing mortality rates. However, due to ethical, cognitive, and medical resource constraints, death autopsy has not been widely adopted. This study identifies potential factors associated with 18.8% (95% CI, 7.2-36.4%) of unexplained child deaths using clinical exome sequencing based on the stored neonatal dried blood spots from newborn screening. It can help these families in conducting salvage molecular autopsies when they have no way to collect samples. At the same time, the results also indicated 28.1% (95% CI, 13.7-46.7%) of secondary findings. With the accumulation of future genetic databases, these findings may further elucidate the true causes of unexplained child deaths. In summary, our study provides a feasible and effective molecular autopsy method for unexplained child deaths.

## Data Availability

The questionnaire and datasets used are available from the corresponding author on request.
